# 24h-gene variation effect of combined bevacizumab/erlotinib in advanced non-squamous non-small cell lung cancer using exon array blood profiling

**DOI:** 10.1186/s12967-017-1174-z

**Published:** 2017-03-30

**Authors:** Florent Baty, Markus Joerger, Martin Früh, Dirk Klingbiel, Francesco Zappa, Martin Brutsche

**Affiliations:** 1 Department of Pulmonary Medicine, Cantonal Hospital St. Gallen, Roschacherstrasse 95, 9007 St. Gallen, Switzerland; 2 Department of Medical Oncology and Hematology, Cantonal Hospital St. Gallen, Roschacherstrasse 95, 9007 St. Gallen, Switzerland; 3 Swiss Group for Clinical Cancer Research, Effingerstrasse 40, 3008 Bern, Switzerland; 4 Oncology Institute of Southern Switzerland, Ospedale Regionale San Giovanni, 6500 Belinzona, Switzerland

**Keywords:** Non-small cell lung cancer, Combined targeted therapies, Blood predictive markers, Exon arrays

## Abstract

**Background:**

The SAKK 19/05 trial investigated the safety and efficacy of the combined targeted therapy bevacizumab and erlotinib (BE) in unselected patients with advanced non-squamous non-small cell lung cancer (NSCLC). Although activating EGFR mutations were the strongest predictors of the response to BE, some patients not harboring driver mutations could benefit from the combined therapy. The identification of predictive biomarkers before or short after initiation of therapy is therefore paramount for proper patient selection, especially among EGFR wild-types. The first aim of this study was to investigate the early change in blood gene expression in unselected patients with advanced non-squamous NSCLC treated by BE. The second aim was to assess the predictive value of blood gene expression levels at baseline and 24h after BE therapy.

**Methods:**

Blood samples from 43 advanced non-squamous NSCLC patients taken at baseline and 24h after initiation of therapy were profiled using Affymetrix’ exon arrays. The 24h gene dysregulation was investigated in the light of gene functional annotations using gene set enrichment analysis. The predictive value of blood gene expression levels was assessed and validated using an independent dataset.

**Results:**

Significant gene dysregulations associated with the 24h-effect of BE were detected from blood-based whole-genome profiling. BE had a direct effect on “Pathways in cancer”, by significantly down-regulating genes involved in cytokine–cytokine receptor interaction, MAPK signaling pathway and mTOR signaling pathway. These pathways contribute to phenomena of evasion of apoptosis, proliferation and sustained angiogenesis. Other signaling pathways specifically reflecting the mechanisms of action of erlotinib and the anti-angiogenesis effect of bevacizumab were activated. The magnitude of change of the most dysregulated genes at 24h did not have a predictive value regarding the patients’ response to BE. However, predictive markers were identified from the gene expression levels at 24h regarding time to progression under BE.

**Conclusions:**

The 24h-effect of the combined targeted therapy BE could be accurately monitored in advanced non-squamous NSCLC blood samples using whole-genome exon arrays. Putative predictive markers at 24h could reflect patients’ response to BE after adjusting for their mutational status.

*Trial registration* ClinicalTrials.gov: NCT00354549

**Electronic supplementary material:**

The online version of this article (doi:10.1186/s12967-017-1174-z) contains supplementary material, which is available to authorized users.

## Background

Combined targeted therapies represent novel therapeutic approaches simultaneously acting on several specific molecular pathways in cancer and having a number of advantages over standard single-targeted agents [1, 2].

Several trials have shown the beneficial effect of epidermal growth factor receptor tyrosine kinase inhibitors (EGFR-TKIs) in advanced non-small cell lung cancer patients (NSCLC) harboring activating EGFR mutations leading to the adoption of EGFR-TKI as standard treatment in this population [3, 4]. Preclinical studies suggested that the combination of an EGFR-TKI together with an angiogenesis inhibitor (e.g. targeting the vascular endothelial growth factor VEGF) can have a synergistic effect [5, 6]. Recent clinical trials showed superior efficacy of the combined anti-angiogenesis bevacizumab (B) with the TKI erlotinib (E) in EGFR mutated patients compared to E alone [7, 8]. More specifically, these trials showed that first line treatments combining BE improved the progression free survival (PFS)—but not overall survival (OS)—of patients harboring an EGFR driver mutation in comparison with E alone [7, 9].

In unselected patients, BE had better PFS than E alone without improvement of survival in recurrent NSCLC suggesting moderate activity of BE [6]. As first line therapy in unselected patients, the overall response rate of BE was 12%, whereas PFS was 3.5 months, again showing moderate activity [10]. Despite the favorable toxicity profile of BE, these results are inferior to chemotherapy first line or immunotherapy second line. The SAKK 19/05 trial from the Swiss Group for Clinical Cancer Research showed that first-line combined BE treatment followed by chemotherapy regimen is feasible with acceptable toxicity and activity in an unselected advanced non-squamous NSCLC population [11]. On the other hand, the phase II TASK study did not show a benefit in terms of PFS for the combination BE in unselected first line advanced non-squamous NSCLC compared with chemotherapy plus B [10, 12].

Although the presence of EGFR mutations is the strongest predictor of the response to anti-EGFR-TKI, a recent meta-analysis showed that wild-type EGFR patients can benefit from the therapy with an improved OS compared with placebo or standard chemotherapy [hazard ratio $=$ 0.780 (95% CI 0.654–0.930)] [13]. Therefore, the identification of very early predictive markers of multiple targeted therapies and the understanding of their mechanisms of action in advanced non-squamous NSCLC is of paramount importance in order to better identify subsets of patients who may still benefit from these treatments.

Blood-based biomarkers in NSCLC are of particular interest as they can be easily and non-invasively accessed [14]. Whole-genome exon arrays provide an ideal platform for the discovery of novel putative biomarkers by investigating expression variations at an exon-level resolution [15]. More specifically, exon arrays allow analyses both at the gene and at the exon level. Exon-level analyses are usually performed to detect alternative splicing events [16].

The aim of the current study was to analyze blood-level exon array profiling data from unselected patients with advanced non-squamous NSCLC before and 24h after initiation of the combined targeted therapy BE. The specific objectives are twofold: (1) uncover which genes from whole blood circulating RNAs are immediately impacted by the effect of the combined therapy BE, and (2) assess the predictive value of these dysregulations.

## Methods

### Lung cancer dataset

**Fig. 1:**
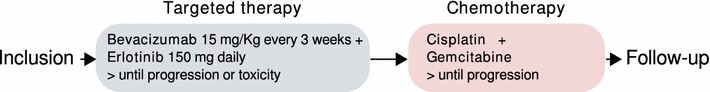
Treatment scheme of the SAKK 19/05 trial. Patients received BE until progression or unacceptable toxicity. Upon disease progression, patients received standard chemotherapy with cisplatin and gemcitabine

The gene expression data set originated from a translational substudy of the phase II SAKK 19/05 trial from the Swiss Group for Clinical Cancer Research (ClinicalTrials.gov: NCT00354549). In the original study, 103 unselected patients with advanced non-squamous NSCLC were enrolled, among which 101 were evaluable. The experimental design of the trial SAKK 19/05 is summarized in Fig. 1. Patients were treated using the combined targeted therapy BE until disease progression or unacceptable toxicity. At progression, standard platinum-based chemotherapy (CT) was used. The primary endpoint of this trial was disease stabilization 12 weeks after initiation of therapy. Secondary endpoints included tumor shrinkage at 12 weeks (TS12), time to progression under BE (TTPBE), time to progression under CT (TTPCT) and OS. Further detailed information about this trial can be found in previous publications [11, 17, 18]. As part of a translational substudy, blood samples were taken at baseline and 24h after initiation of treatment for gene expression analysis in a subset of 49 patients. The current study was approved by the ethics committee of the canton St. Gallen (EKSG 06/012).

### Exon array analysis

RNA from whole blood samples was extracted and quality checked. Six pairs of samples had to be excluded from the analysis due to low quality, whereas RNA extracts provided sufficient quality for microarray hybridization in 43 out of 49 pairs of sample. Messenger RNAs were hybridized on Affymetrix Human Exon 1.0 ST arrays (Affymetrix, Santa Clara, CA, USA) following standard recommendations from the manufacturer. This microarray platform measures genome-wide exon-level expression in over 1.4 million probe sets, and allows the investigation of genomic variations both at the gene and at the exon level. For the sake of this analysis, 439,778 exonic probe sets (within 38,900 genes) were kept in the analysis, after filtering out intronic, intergenic and unreliable probe sets (according to the nomenclature defined in the R package annmap [19]). Raw data (Affymetrix CEL files) have been deposited in NCBI’s Gene Expression Omnibus (GEO), and are accessible through GEO Series accession number GSE61676. The exon level probe sets were pre-processed, quality checked and normalized using the RMA procedure (including background correction, quantile normalization and median-polish summarization) as implemented in the R package oligo [20, 21].

### Statistical considerations

**Fig. 2:**
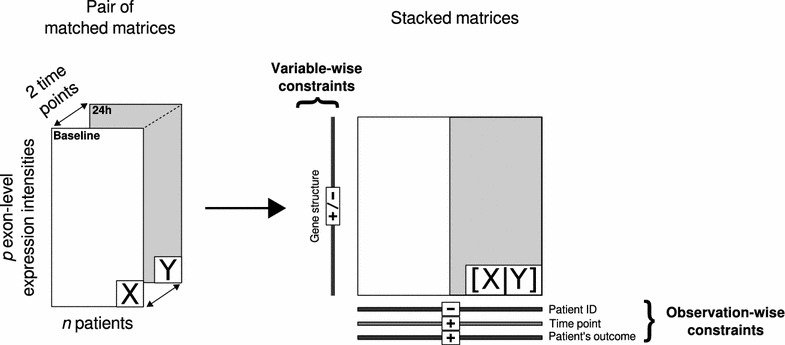
Design of experiment and scheme of dually constrained correspondence analysis. Two matched tables $\mathbf {X}$ and $\mathbf {Y}$ are analyzed by DCCA. The 2 tables are rearranged into one stacked table. Additional external information on both rows and columns are used as positive and/or negative constraints

The current experimental design includes a repeated measurement of genome-wide exon-level expressions in 43 patients at two different time points (baseline and 24h after initiation of therapy). The exon expression data are stored in a pair of fully matched tables. Dually constrained correspondence analysis (DCCA)—an extension of the multivariate technique correspondence analysis—was used to investigate the 24h-change in exon expression levels [16]. DCCA uses observation- and variable-wise linear constraints in order to take into account complex experimental designs including within-patient repeated measurements and variables grouped into hierarchical levels (within-gene exonic structures). The theoretical scheme of DCCA as applied to our experimental design is summarized in Fig. 2. The mathematical underpinnings of DCCA are further described in Additional file 1. In the current work, the following parametrization of DCCA was used: (1) the two fully matched tables of exon-level expression intensities at baseline and 24h were stacked observation-wise; (2) an observation-wise between-time constraint (baseline vs. 24h) was applied after partialling out the within-patient effect; (3) a variable-wise constraint indicating the within-gene exonic structure was applied.

Generalized linear (mixed effects) models were used in order to test the predictive value of the identified biomarkers. Binary endpoints were tested using logistic regression, time to event data were modeled using Cox proportional hazards regression, and continuous variables were modeled using (multiple) linear regression. Mixed effects modeling was used when testing associations in the frame of the within-patient repeated measurement design. The adjustment for patients’ EGFR mutational status was done by including the mutational status as covariate in the predictive models. The significance level used for the discovery of the putative predictive markers was set to 0.001. Gene signatures combining the information of the best predictive candidates were built using the metagene approach [22]. In this approach, the linear combination of several genes is calculated as follows: (1) The matrix of normalized gene expression intensities for all patients at a given time point (rows) and for all candidate genes (columns) is analyzed using unscaled principal component analysis (PCA); (2) The row coordinates (scores) on the first PCA axis summarizing the largest amount of variance are extracted (metagene score); (3) Based on the median of the metagene score, a binary score is created to discriminate between low versus high-risk patients.

### Computational considerations

All analyses were implemented using the R statistical software [23] including the extension package ade4 [24], as well as dedicated packages from the Bioconductor project [25] such as the package oligo for microarray preprocessing [21] and the annotation package annmap (*Homo sapiens* database version 86). Functions available in ade4 (including dudi.coa, wca, bca, pcaiv and pcaivortho) can theoretically be used to carry out DCCA. However, for reasons of computational efficiency due to the extensive size of exon array datasets, the DCCA algorithm was substantially optimized and a new R extension package dcca is available (see Additional file 1). Hypothesis testing for the identification of predictive biomarkers was carried out using the following R packages: lme4, coxme and multcomp.

### Gene functional annotations and validations

Gene set enrichment analysis was done by interrogating the molecular knowledge databases Kyoto Encyclopedia of Genes and Genomes (KEGG) [26] and WikiPathways [1] using the functional annotation web-service WebGestalt [27]. Enrichment analyses were based on the list of 100 most dysregulated genes (as identified by DCCA), as well as the lists of genes which were significantly predicting patient’s outcome (each of the investigated endpoints). The significance of the enrichment was obtained using hypergeometric tests. Validation was carried out using the lung data set from the Kaplan–Meier Plotter (KMplotter) web tool [28]. KMplotter is a manually curated database including gene expression level information about more than 50,000 Affymetrix probe set IDs together with associated clinical information. The prognostic value of single or multiple genes can be assessed with regard to relapse free and overall survival. Another independent lung cancer dataset was used for external validation. This gene expression microarray dataset includes 85 lung adenocarcinoma tumor samples and is part of the program “Carte d’Identité des Tumeurs” (CIT) from the french national cancer league [29]. Samples were profiled using the Affymetrix Human Genome U133 Plus 2.0 Array and raw data are available in NCBI’s Gene Expression Omnibus through GEO Series accession number GSE30219. Furthermore, the results were discussed in the light of available literature findings.

## Results

### Patients characteristics

The characteristics of the 43 patients are reported in Table 1. Patients were late stage (91% stage IV/9% stage IIIb) non-squamous NSCLC. Five out of 43 patients had demonstrable EGFR mutations: one on exon 18 (E709A-G719S), three on exon 19 (Del L747-G749, Del E746-A750 and R748-S752) and one on exon 21 (L858R). The median age was 61 years old (IQR 54–66) and the sex ratio was 0.44 (19 males/24 females). Disease stabilization at 12 weeks was reached in 53% of patients. The median tumor shrinkage at 12 weeks was 15.8%. The median overall survival was 11.1 (95% CI 10.1–17.9) months. The median time-to-progression under BE was 4.0 (95% CI 2.8–6.0) months, whereas the median time-to-progression under CT was 2.6 (95% CI 1.7–5.7) months.

**Table 1:** Patients characteristics

Variables	
Number of patients (*n*)	43
Age (median [range])	61 (35–78)
Gender (# male [%])	19 (44.2%)
Stage (*n* [%])	IIIb: 4 (9.3%); IV: 39 (90.7%)
Demonstrable EGFR mutations (*n* [%])	5 (11.6%)
Disease stabilization at 12 weeks (*n* [%])	23 (53.5%)
Tumor shrinkage at 12 weeks (median [IQR]), in % of tumor size at baseline	15.8% (−2.5 to 26.2%)
Median overall survival (95% CI), in months	11.1 (10.1–17.9)
Median time-to-progression under BE (95% CI), in months	4.0 (2.8–6.0)
Median time-to-progression under CT (95% CI), in months	2.6 (1.7–5.7)

The table summarizes the characteristics of the 43 patients included in the study

### 24h gene dysregulation

The 100 genes mostly dysregulated (54 up-regulated vs. 46 down-regulated) by the 24h effect of BE are summarized in Table  2. The genes were involved in all aspects of tumor biology. This includes genes involved in mitosis and cell cycle processes such as the cancer susceptibility candidate 5 (CASC5) encoding for a protein influencing the spindle assembly checkpoint during eukaryotic cell cycle; the centromere-associated protein E (CENPE) which accumulates and play a key stabilizing role during mitosis; the protein furry homolog (FRY) which plays a crucial role in the structural integrity of mitotic centrosomes; kinetochore associated 1 (KNTC1) encoding for a protein ensuring proper chromosome segregation during cell division; Phospholipase D1 (PLD1) involved in cancer progression [30] and in the regulation of mitosis in relationship with the “Ras signaling pathway” and “Pathways in cancer”. Other dysregulated genes were involved in energy-dependent metabolisms (ATPase/GTPase). This includes genes from the ATPase Family (ATAD2) known to be related to gastric cancer network, and which may play an important role in cell proliferation and cell cycle progression of breast cancer cells; Guanylate Binding Protein 4 (GBP4) related to GTPase activity and associated with the interferon signaling pathway; dedicator of cytokinesis 10 (DOCK10) acting on GTPase and related to hemostasis and regulation of cell division cycle 42 (CDC42) activity. Mechanisms of cell migration are also controlled by a series of dysregulated genes such as ADAM Metallopeptidase Domain 19 (ADAM19) regulating cell migration, cell adhesion and cell-cell/cell-matrix interactions, supposed to play an important role in pathological processes including cancer; BMX non-receptor tyrosine kinase (BMX) encoding for a protein implicated in several signal transduction pathways regulating tumorigenicity of cancer cells; Epidermal Growth Factor Receptor Pathway Substrate 8 (EPS8) encoding for a protein having functions in part of the EGFR pathway and being related to Tyrosine Kinases/Adaptors and Development FGFR signaling pathways; Fms-Related Tyrosine Kinase 3 (FLT3) encoding for a class III receptor tyrosine kinase regulating hematopoiesis, and whose action is related to apotosis, proliferation and differentiation processes; Interleukin 1 Receptor, Type II (IL1R2) controling many cellular functions including proliferation, differentiation, and cell survival/apoptosis; Matrix Metallopeptidase 9 (MMP9) involved in tissue/matrix remodeling, playing a central role in cell proliferation, migration, differentiation, showing an altered expression in a number of different human cancers with poor prognosis. Apoptosis was regulated through the action of various genes including baculoviral IAP repeat containing 3 (BIRC3) encoding for an inhibitor of apoptosis protein acting on killing tumor cells; death-associated protein kinase 2 (DAPK2) whose overexpression was shown to induce cell apoptosis; Retinoblastoma-Like 1 (RBL1) encoding for a tumor suppressor protein involved in cell cycle regulation; insulin-like growth factor 1 receptor (IGF1R) encoding for a growth factor with tyrosine kinase activity, having an anti-apoptotic effect and being highly overexpressed in most malignant tissues [31].

**Table 2:** List of the 100 most dysregulated genes due to the 24h effect of BE

Ensembl	Gene symbol	Description	Gene dysregulation at 24h
ENSG00000125257	ABCC4	ATP-binding cassette, sub-family C (CFTR/MRP), member 4	Up-regulated
ENSG00000114770	ABCC5	ATP-binding cassette, sub-family C (CFTR/MRP), member 5	Down-regulated
ENSG00000173208	ABCD2	ATP-binding cassette, sub-family D (ALD), member 2	Up-regulated
ENSG00000151726	ACSL1	Acyl-CoA synthetase long-chain family member 1	Down-regulated
ENSG00000135074	ADAM19	ADAM metallopeptidase domain 19 (meltrin beta)	Down-regulated
ENSG00000154027	AK5	Adenylate kinase 5	Up-regulated
ENSG00000151150	ANK3	Ankyrin 3, node of Ranvier (ankyrin G)	Up-regulated
ENSG00000206560	ANKRD28	Ankyrin repeat domain 28	Up-regulated
ENSG00000118520	ARG1	Arginase, liver	Down-regulated
ENSG00000196914	ARHGEF12	Rho guanine nucleotide exchange factor (GEF) 12	Up-regulated
ENSG00000156802	ATAD2	ATPase family, AAA domain containing 2	Up-regulated
ENSG00000085224	ATRX	Alpha thalassemia/mental retardation syndrome X-linked (RAD54 homolog, *S. cerevisiae*)	Up-regulated
ENSG00000023445	BIRC3	baculoviral IAP repeat-containing 3	Up-regulated
ENSG00000102010	BMX	BMX non-receptor tyrosine kinase	Down-regulated
ENSG00000136492	BRIP1	BRCA1 interacting protein C-terminal helicase 1	Up-regulated
ENSG00000197603	C5orf42	Chromosome 5 open reading frame 42	Up-regulated
ENSG00000152495	CAMK4	Calcium/calmodulin-dependent protein kinase IV	Up-regulated
ENSG00000137812	CASC5	Cancer susceptibility candidate 5	Up-regulated
ENSG00000138778	CENPE	Centromere protein E, 312 kDa	Up-regulated
ENSG00000198707	CEP290	Centrosomal protein 290 kDa	Up-regulated
ENSG00000106034	CPED1	Cadherin-like and PC-esterase domain containing 1	Up-regulated
ENSG00000103196	CRISPLD2	Cysteine-rich secretory protein LCCL domain containing 2	Down-regulated
ENSG00000146122	DAAM2	Dishevelled associated activator of morphogenesis 2	Down-regulated
ENSG00000035664	DAPK2	Death-associated protein kinase 2	Down-regulated
ENSG00000137628	DDX60	DEAD (Asp-Glu-Ala-Asp) box polypeptide 60	Up-regulated
ENSG00000174485	DENND4A	DENN/MADD domain containing 4A	Up-regulated
ENSG00000135905	DOCK10	Dedicator of cytokinesis 10	Up-regulated
ENSG00000147459	DOCK5	Dedicator of cytokinesis 5	Down-regulated
ENSG00000088387	DOCK9	Dedicator of cytokinesis 9	Up-regulated
ENSG00000178904	DPY19L3	Dpy-19-like 3 (*C. elegans*)	Down-regulated
ENSG00000134765	DSC1	Desmocollin 1	Up-regulated
ENSG00000135636	DYSF	Dysferlin, limb girdle muscular dystrophy 2B (autosomal recessive)	Down-regulated
ENSG00000198919	DZIP3	DAZ interacting protein 3, zinc finger	Up-regulated
ENSG00000102189	EEA1	Early endosome antigen 1	Up-regulated
ENSG00000151491	EPS8	Epidermal growth factor receptor pathway substrate 8	Up-regulated
ENSG00000089048	ESF1	ESF1, nucleolar pre-rRNA processing protein, homolog (*S. cerevisiae*)	Up-regulated
ENSG00000198734	F5	Coagulation factor V (proaccelerin, labile factor)	Down-regulated
ENSG00000140525	FANCI	Fanconi anemia, complementation group I	Up-regulated
ENSG00000138829	FBN2	Fibrillin 2 (congenital contractural arachnodactyly)	Down-regulated
ENSG00000139132	FGD4	FYVE, RhoGEF and PH domain containing 4	Down-regulated
ENSG00000122025	FLT3	Fms-related tyrosine kinase 3	Down-regulated
ENSG00000161791	FMNL3	Formin-like 3	Up-regulated
ENSG00000073910	FRY	Furry homolog (*Drosophila*)	Down-regulated
ENSG00000162654	GBP4	Guanylate binding protein 4	Up-regulated
ENSG00000182885	GPR97	G protein-coupled receptor 97	Down-regulated
ENSG00000106070	GRB10	Growth factor receptor-bound protein 10	Down-regulated
ENSG00000084110	HAL	Histidine ammonia-lyase	Down-regulated
ENSG00000120694	HSPH1	Heat shock 105/110 kDa protein 1	Up-regulated
ENSG00000140443	IGF1R	Insulin-like growth factor 1 receptor	Down-regulated
ENSG00000197081	IGF2R	Insulin-like growth factor 2 receptor	Down-regulated
ENSG00000115604	IL18R1	Interleukin 18 receptor 1	Down-regulated
ENSG00000115594	IL1R1	Interleukin 1 receptor, type I	Down-regulated
ENSG00000115590	IL1R2	Interleukin 1 receptor, type II	Down-regulated
ENSG00000109452	INPP4B	Inositol polyphosphate-4-phosphatase, type II, 105 kDa	Up-regulated
ENSG00000102445	KIAA0226L	KIAA0226-like	Down-regulated
ENSG00000137261	KIAA0319	KIAA0319	Down-regulated
ENSG00000110318	KIAA1377	KIAA1377	Up-regulated
ENSG00000054523	KIF1B	Kinesin family member 1B	Down-regulated
ENSG00000138182	KIF20B	Kinesin family member 20B	Up-regulated
ENSG00000139116	KIF21A	Kinesin family member 21A	Up-regulated
ENSG00000068796	KIF2A	Kinesin heavy chain member 2A	Up-regulated
ENSG00000184445	KNTC1	Kinetochore associated 1	Up-regulated
ENSG00000126777	KTN1	Kinectin 1 (kinesin receptor)	Up-regulated
ENSG00000123384	LRP1	Low density lipoprotein-related protein 1 (alpha-2-macroglobulin receptor)	Down-regulated
ENSG00000186205	MARC1	Mitochondrial amidoxime reducing component 1	Down-regulated
ENSG00000257335	MGAM	Maltase-glucoamylase (alpha-glucosidase)	Down-regulated
ENSG00000171843	MLLT3	Myeloid/lymphoid or mixed-lineage leukemia (trithorax homolog, *Drosophila*); translocated to, 3	Up-regulated
ENSG00000196549	MME	Membrane metallo-endopeptidase	Down-regulated
ENSG00000100985	MMP9	Matrix metallopeptidase 9 (gelatinase B, 92 kDa gelatinase, 92 kDa type IV collagenase)	Down-regulated
ENSG00000051825	MPHOSPH9	M-phase phosphoprotein 9	Up-regulated
ENSG00000138119	MYOF	Myoferlin	Up-regulated
ENSG00000049759	NEDD4L	Neural precursor cell expressed, developmentally down-regulated 4-like	Up-regulated
ENSG00000184613	NELL2	NEL-like 2 (chicken)	Up-regulated
ENSG00000173145	NOC3L	Nucleolar complex associated 3 homolog (*S. cerevisiae*)	Up-regulated
ENSG00000179299	NSUN7	NOL1/NOP2/Sun domain family, member 7	Down-regulated
ENSG00000111581	NUP107	Nucleoporin 107 kDa	Up-regulated
ENSG00000159339	PADI4	Peptidyl arginine deiminase, type IV	Down-regulated
ENSG00000123836	PFKFB2	6-phosphofructo-2-kinase/fructose-2,6-biphosphatase 2	Down-regulated
ENSG00000075651	PLD1	Phospholipase D1, phosphatidylcholine-specific	Down-regulated
ENSG00000101868	POLA1	Polymerase (DNA directed), alpha 1	Up-regulated
ENSG00000080839	RBL1	Retinoblastoma-like 1 (p107)	Up-regulated
ENSG00000257743	RP11-1220K2.2	Putative inactive maltase-glucoamylase-like protein LOC93432	Down-regulated
ENSG00000226891	RP11-182I10.3	Uncharacterized LOC101927084	Down-regulated
ENSG00000258476	RP11-76E17.3	NA	Down-regulated
ENSG00000140386	SCAPER	S phase cyclin A-associated protein in the ER	Up-regulated
ENSG00000018280	SLC11A1	Solute carrier family 11 (proton-coupled divalent metal ion transporters), member 1	Down-regulated
ENSG00000140090	SLC24A4	Solute carrier family 24 (sodium/potassium/calcium exchanger), member 4	Down-regulated
ENSG00000112053	SLC26A8	Solute carrier family 26, member 8	Down-regulated
ENSG00000157800	SLC37A3	Solute carrier family 37 (glycerol-3-phosphate transporter), member 3	Down-regulated
ENSG00000136824	SMC2	Structural maintenance of chromosomes 2	Up-regulated
ENSG00000163029	SMC6	Structural maintenance of chromosomes 6	Up-regulated
ENSG00000009694	TENM1	Teneurin transmembrane protein 1	Down-regulated
ENSG00000169902	TPST1	Tyrosylprotein sulfotransferase 1	Down-regulated
ENSG00000198677	TTC37	Tetratricopeptide repeat domain 37	Up-regulated
ENSG00000155657	TTN	Titin	Up-regulated
ENSG00000120800	UTP20	UTP20, small subunit (SSU) processome component, homolog (yeast)	Up-regulated
ENSG00000197969	VPS13A	Vacuolar protein sorting 13 homolog A (S. cerevisiae)	Up-regulated
ENSG00000163625	WDFY3	WD repeat and FYVE domain containing 3	Down-regulated
ENSG00000213799	ZNF845	Zinc finger protein 845	Up-regulated
ENSG00000167232	ZNF91	Zinc finger protein 91	Up-regulated

Information includes the Ensembl code, the gene symbol, description and sign of dysregulation. For convenience, the genes are ordered alphabetically

Based on the 100 best candidates identified by DCCA, three main pathways (according to the KEGG functional annotation database) were significantly altered by the 24h effect of the combined therapy BE: Hematopoietic cell lineage (KEGG pathway hsa04640; $p=0.0094$); ABC transporters (KEGG pathway hsa02010; $p=0.0085$); Pathways in cancer (KEGG pathway hsa05200; $p=0.0204$).

**Fig. 3:**
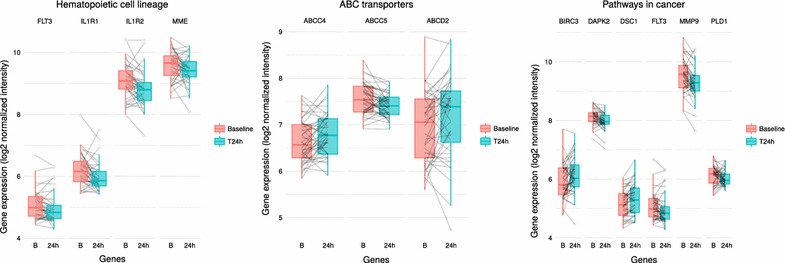
Boxplot representation of the gene expression levels (logarithm base 2 normalized intensity) before (B) and 24h after initiation of bevacizumab/erlotinib in the KEGG pathways “Hematopoietic cell lineage” (hsa04640), “ABC transporters” (hsa02010), and “Pathways in cancer” (hsa05200). The genes depicted in this representation belong the list of the 100 most dysregulated genes

**Fig. 4:**
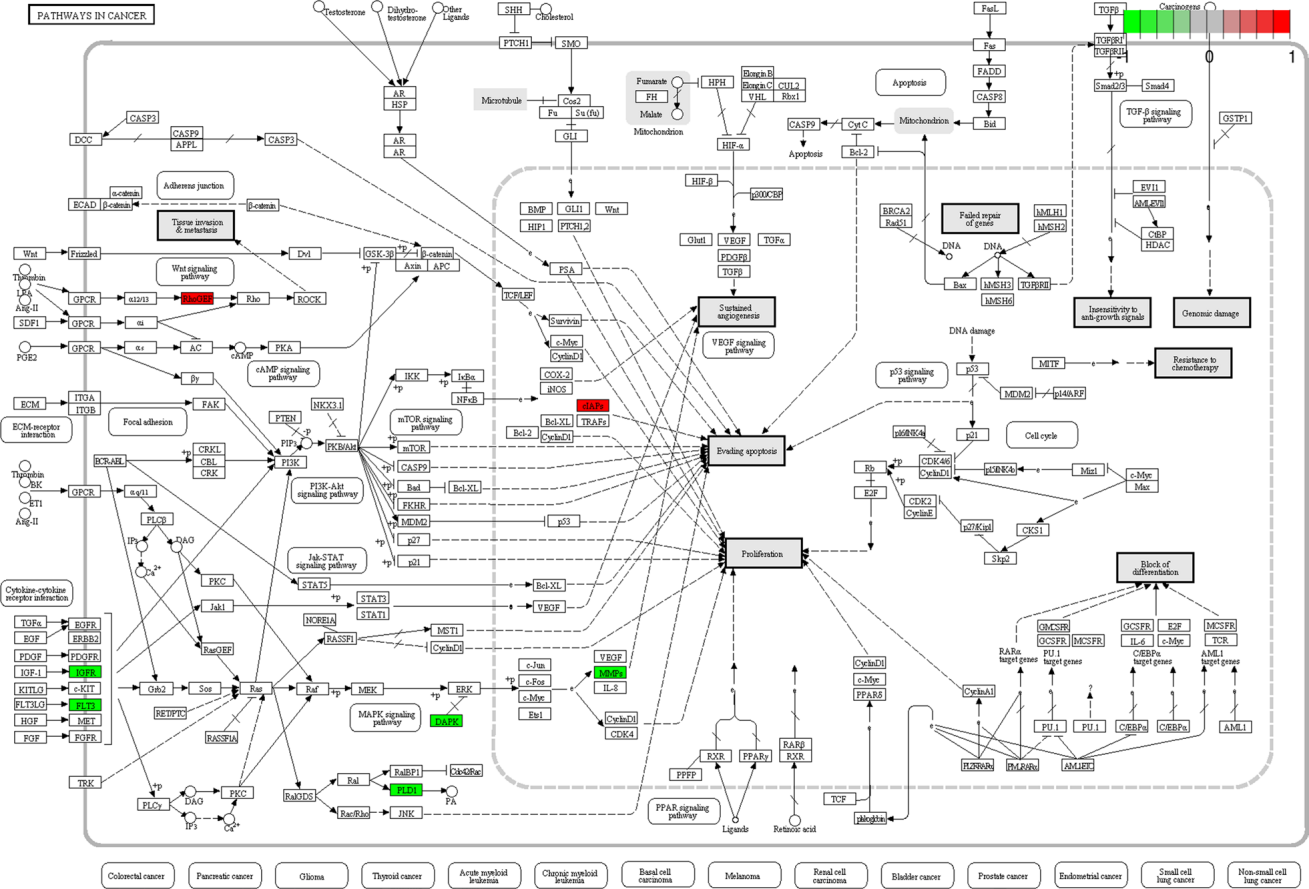
KEGG pathway hsa05200 “Pathways in cancer”. Genes highlighted in *red* and *green* were up-regulated and down-regulated due to the 24h action of bevacizumab/erlotinib, respectively

Figure 3 displays the gene expression levels of each genes (baseline and 24h) within these dysregulated pathways. Figure  4 shows the detailed KEGG’s “Pathways in Cancer” highlighting the down-regulated genes within the sub-pathways “Cytokine–cytokine receptor information” and “MAPK signaling pathways”. The down-regulation of these sub-pathways contributes to the limitation of cell proliferation.

These pathway dysregulations are well in line with the expected effects of both erlotinib (“Pathways in cancer”; “ABC transporters”) and bevacizumab (“Hematopoietic cell lineage”). “Pathways in cancer” is a generic pathway, including genes involved in various aspects of tumorigenesis such as phenomena of proliferation, invasion, resistance and apoptosis. Several diseases are inter-related to this pathway, including non-small cell lung cancer. Most genes from the “Pathways in cancer” (FLT3, IGF1R, DAPK2, PLD1 and MMP9) are inhibited due to the action of BE resulting in an anti-proliferative and pro-apoptotic action of the combined therapy, with the notable exception of the up-regulation of the apoptosis inhibitor BIRC3. The combined action of BE results in the inhibition of all genes that belong to the “Hematopoietic cell lineage” (FLT3, IL1R1, IL1R2, MME). Hematopoietic stem cells play important roles for angiogenesis [32]. The down-regulation of genes within the pathway “Hematopoietic cell lineage” may be related to the specific anti-angiogenic action of bevacizumab [33, 34]. On the other hand, the dysregulation of genes that belong to the pathway “ABC transporters” is probably associated with the energy-related mechanisms of action of erlotinib [35, 36].

Gene set enrichment analysis based on the WikiPathways database provides information on additional cancer- and energy-related activated pathways including “gastric cancer network 2” (WP2363, $p=0.010$), “IL1 megakaryocytes in obesity” (WP2865, $p=0.006$), “apoptosis modulation and signaling” (WP1772, $P=0.011$) and “gastric cancer network 1” (WP2361, $p=0.009$).

### Gene expression predictive value

The predictive value of the genes that were mainly dysregulated due to the 24h effect of BE was investigated. The following endpoints were considered: disease stabilization at 12 weeks, tumor shrinkage at 12 weeks, time to progression under BE, time to progression under chemotherapy, and overall survival. The magnitude of the 24h change in expression of the 100 most dysregulated genes was not significantly associated with any of the investigated endpoints, after adjustment for the patients mutational status.

Table 3 summarizes the predictive value of the blood gene expression at baseline and 24h after initiation of BE. Putative markers at baseline that predicted patient’s overall survival included Cancer susceptibility candidate 1 (CASC1). At baseline, 142 genes were identified as putative predictive markers of tumor shrinkage at 12 weeks. Among those genes, there was a significant enrichment of the KEGG signaling pathways “Phagosome” (hsa04145) and “Protein digestion and absorption” (hsa04974).

**Table 3:** Putative predictive markers of the patient’s response to bevacizumab/erlotinib

Time	Endpoint^a^	Gene symbols	KEGG pathway enrichment
Baseline	TS12	ANO2, VPS13D, EML1, C22orf31, FERMT1, SFRP4, SUPT6H, GYS2, THBS4, ATP6V1B1, TCF21, ESRRB, NEUROD4, SOX15, GATA5, PRRC2B, SLC7A10, LOXL4, DISP2, TRIM7, DRD2, GPHA2, KCTD15, PAQR4, ANKZF1, SHROOM1, FABP7, FBXL21, KCNK5, MBL2, LY6D, VASN, FSTL5, MECP2, ROR2, TPT1-AS1, DCDC1, HS6ST2, HSPB7, NWD2, OR5M3, SLC35E3, UNC119B, OR10AB1P, UMODL1, ERN1, RNF151, CALHM1, TMPRSS12, KLK12, KRT14, LINC01551, NWD1, C15orf52, IL1RAPL2, TRRAP, TPK1, MYO18A, DSCR8, B3GNT6, RNA5SP430, C1orf132, PPAPDC1A, BTBD17, LAYN, CST9LP1, ST8SIA6-AS1, AL592528.1, RP11-337C18.4, GAPDHP44, HAUS7, AC010904.1, CELA3B, RP1-288M22.1, U3, CTA-929C8.7, ARSEP1, UBE2D3P4, RP11-38C18.2, RP11-94M14.2, AC007312.3, LINC00237, RP11-66N5.2, LINC01375, C1DP4, RP11-20P5.2, RP5-888M10.2, AC105443.2, FGFR1OP2P1, RP5-855F14.2, TFAMP1, POLR3KP1, CRYGFP, TBC1D3P7, RP11-336N8.1, DCUN1D2-AS, RP11-528G1.2, ZBTB22, TSPY15P, RP11-98G13.1, AC007679.4, KCNQ1DN, RN7SL549P, SMKR1, PNMA2, PI4KA, RP11-520P18.1, RP11-572M11.3, OR10J7P, RP11-331K21.1, RP11-340A13.1, LINC00977, RP3-368B9.2, SNORA18, HNRNPCP8, MPV17L2, NAV2-AS5, CTD-2517M22.14, ENPP7P5, RP11-150C16.1, HNRNPA3P10, RP11-316E14.2, RP11-566K19.5, RP11-521O16.1, RP11-616M22.7, RP11-523L20.2, AC009120.4, RP11-106M3.3, RP11-553K8.5, RP11-189E14.4, RP11-520P18.5, SAMD11P1, RP11-286N3.2, SNRPCP4, RP11-343K8.3, AC005307.3, AC010524.4, CTB-31C7.3, WI2-80269A6.1, RP11-401N16.1, RP11-416H1.1, RP11-1072C15.7	Phagosome (hsa04145, $p=0.052$) Protein digestion and absorption (hsa04974, $p=0.058$)
	DS12	–	–
	OS	CASC1, HIST1H1A, FAM86C2P, Y_RNA, IGHV5-51, RP11-174G6.1, HNRNPA1P63, EEF1B2P1, RP3-403L10.3, RPL7P53, IGKV1-5, MRPS36P2, RP11-415I12.3, RNU6-412P, RNU6-1224P, RP11-61G23.2, CTD-2547H18.1, RP11-295G12.1, ZNF23	–
	TTPBE	BDKRB1, NRN1, LAMC1, ZNF462, LRRC43, SGSM1, FSCN2, C9orf92, RNU6-83P, RPL7AP14, RP11-793K1.1, RP3-433F14.1, RP1-292B18.4, AC098592.7, AC114812.8, TOMM20P1, LL22NC03-88E1.17, C12orf77, RP1-213J1P__B.1, LINC01038, MARK2P12, XRCC6P3, SHC1P1, RP3-463P15.1, LL0XNC01-116E7.2, COL4A2-AS1, CYCSP44, RAC1P8, SLMO2-ATP5E, AF127577.12, STARD13-AS, RN7SL797P, RN7SL598P, RP11-544A12.8, RP11-415I12.3, RP11-61G23.2, RP11-6B19.3, RP11-136F16.2, CASC18, AC002306.1, RP11-552E10.1, RP11-361M10.3, RP11-203B7.2, RP11-475B2.1, RP11-763E3.1, RP13-516M14.2, RP11-820I16.1	–
	TTPCT	KRTAP2-3, AC090957.2, KALP, RP11-157I4.4, TSIX	–
24h after initiation of BE	TS12	PDZD4, SH3BP1, CALD1, SPX, MAPKBP1, MMRN1, *EGF*, PLOD2, ANKRD61, *FZD5*, CLEC1B, SLC39A13, SMCO4, BCRP2, TSNARE1, TDRP, TOP1MT, TPTE2P3, RNU105C, Y_RNA, RN7SKP257, AC006988.1, ATP5HP3, RNU6-887P, RP11-361F15.2, BANF1P2, AC099344.2, LINC00884, DDX39BP2, RP11-486M23.1, RP11-167N24.3, RP11-693J15.3, RP11-433P17.3	*Pathways in cancer* (hsa05200, $p=0.031$)
	DS12	–	–
	OS	MYO1C, Y_RNA, DEFB134, Y_RNA, IGLV3-21, IGLC7, IGHA2, IGHA1, IGHV3-15, IGHV3-23, IGHV5-51, SOD1P1, AC016768.1, LINC01032, ZSCAN31, RP11-169N13.4, IGKV1-5, IGHV3-65, IGLC4, RP11-280K24.1	–
	TTPBE	TNMD, TSPAN9, PRICKLE3, *RAD51*, MCM10, TCF3, ATP2A3, OPHN1, FBXL19, PCK2, PLTP, *E2F1*, PCYT1B, CKM, APBA1, CNTNAP1, FOXM1, KIF20A, CIT, HJURP, E2F8, KLF16, APOC1, ATP8B3, EPHB2, CTIF, TICRR, CTU1, CPXCR1, GRIK4, C16orf78, CKB, VKORC1, TK1, METTL7B, MZB1, ZNF296, RRM2, *JUP*, PCP2, CADM2, SLC25A22, KLHL28, GJC1, MARCH11, GAS2L1, *MITF*, HIST1H2BM, SLC25A29, SRC, MYO1C, RNU12-2P, Y_RNA, D86998.1, IGLV6-57, IGLV3-21, IGHG1, IGHJ1, IGHV6-1, IGHV4-28, AP005482.3, RP3-407E4.4, RP11-535M15.1, IPPKP1, FAM195B, RP11-69C17.2, HDGFP1, SCAMP4, RD3L, TUBB4AP1, SDAD1P2, RP11-321L2.1, SPATA31B1P, AC005772.2, RP11-22C8.1, VN1R38P, ITGA9-AS1, NIFK-AS1, B3GNT9, RP11-252M21.6, RN7SL60P, RP11-266N13.2, RP11-517I3.1, RP11-364C11.2, RACGAP1P, RP11-545N8.3, RP11-81A1.3, RP11-2C24.5, AF213884.2, PRKCA-AS1, CTD-2319I12.2	Melanoma (hsa05218, $p=0.029$) Pancreatic cancer (hsa05212, $p=0.029$) PPAR signaling cancer (hsa03320, $p=0.029$) arginine and proline metabolism (hsa00330, $p=0.029$) *Pathways in cancer* (hsa05200, $p=0.029$) Pyrimidine metabolism (hsa00240, $p=0.041$)
	TTPCT	TTTY14, MTND1P4, RP11-875H7.2, LINC01021, AC008565.1, LINC01486	–

Gene significantly predictive of the patient’s response ($p<0.001$) are reported together with the associated enriched KEGG pathways. Genes that belonged to the KEGG pathway “Pathways in cancer” are highlighted in italic

^a^TS12: tumor shrinkage at 12 weeks; DS12: disease stabilization at 12 weeks; OS: overall survival; TTPBE: time-to-progression under bevacizumab/erlotinib; TTPCT: time-to-progression under chemotherapy

Putative predictive markers from blood gene expression at 24h after initiation of treatment of the TTPBE included four genes enriched in the pathway “Pathways in cancer”. These genes were the E2F transcription factor 1 (E2F1), RAD51 recombinase (RAD51), the junction plakoglobin (JUP), and the microphthalmia-associated transcription factor (MITF). E2F1 plays a critical role in the control of cell cycle and acts as a tumor suppressor. E2F1 was found to be associated with phenomena of resistance of targeted therapy in breast cancer [37]. There was also a significant enrichment in the signaling pathway “Pathways in cancer” among the predictors of TS12 at 24h: genes frizzled class receptor 5 (FZD5) and epidermal growth factor (EGF). EGF encodes for a protein playing an important role in the cell growth, proliferation and differentiation. It binds with high affinity epidermal growth factor receptor. Its dysregulation has been associated with cancer progression [38]. Other pathways associated with 24h predictive markers of TTPBE included the cancer-related pathways “Melanoma”, “Pancreatic cancer”, “PPAR signaling cancer”, as well as the metabolism-related pathways “arginine and proline metabolism” and “Pyrimidine metabolism”.

**Fig. 5:**
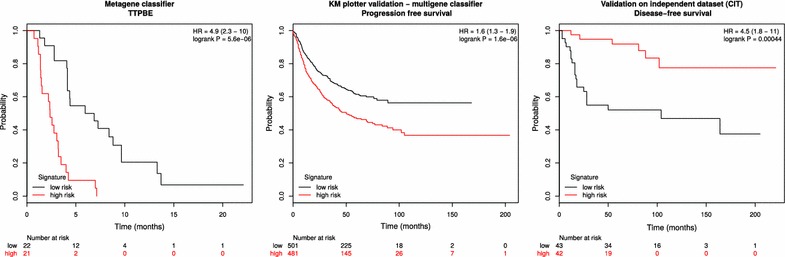
Metagene classifier of time-to-progression under BE. The *left panel* displays the classification of low- versus high-risk patients based on the 91-gene metagene. The *central panel* shows the classification obtained by the KMplotter online validation tool using a multigene classifier. The *right panel* shows the classification obtained by the external CIT validation dataset. Hazard ratios and log rank test *p* values are reported in the *upper right corner* of the each panel

All putative predictive markers of TTPBE at 24h were combined into a 91-gene metagene. Patients could be significantly classified into low-risk versus high-risk according to their median metagene score (HR 4.93 [95% CI 2.34 to 10.39], log-rank test: $p < 0.001$) (Fig. 5, left panel). The median TTPBE were 2.46 (95% CI 1.54–3.22) months vs. 6.87 (95% CI 4.14–13.31) months in the high-risk and low-risk populations, respectively. This finding was successfully validated using the KMplotter web tool (Fig. 5, central panel) and the external CIT validation dataset (Fig. 5, right panel). The predictive value of the metagene remained significant after adjusting for the patient’s mutational status (Cox proportional hazards regression after adjustment for the mutational status: HR 2.63 [95% CI 1.87–3.70], $p < 0.001$). An illustration of selection of responders based on the metagene score is provided in the Results section of the Additioanl file 1.

## Discussion

The analysis of the immediate effect of BE in late stage non-squamous NSCLC reveals a series of important mechanisms dysregulated by the combined action of both therapies. Important activated pathways involved mechanisms such as apoptosis evasion, anti-proliferation and anti-angiogenesis. Interestingly, it was possible to detect these dysregulations directly in the blood, showing that potential biomarkers could be identified at the blood level. The changes measured in the blood over a small time period (24h) were of small magnitude, yet consistent among patients. The use of a within-patient design of experiment including 2 time points before and after treatment helped to characterize these gene variations despite the relatively small sample size.

The choice of the multivariate method DCCA over more common gene-by-gene approaches was driven by the fact that DCCA addresses the problem of the identification of differentially expressed genes (in within-patient repeated measures designs) in a multivariate manner. This is more statisfactory since it allows to take into account potential gene correlations/interactions using a single computationally efficient procedure. DCCA is an exploratory method appropriate for the purpose of the current hypothesis-generating translational study. On the other hand, gene-by-gene approaches are simple and flexible and could be preferred in case of more complex designs or when applied to confirmatory analyses.

Although the magnitude of change of the most dysregulated genes over 24h was not predictive of the patient’s outcome, both the gene expression level at baseline and 24h revealed a series of putative predictive genes. While DS12 was defined as primary clinical endpoint of the original SAKK 19/05 trial, endpoints reflecting the activity of the treatment on the disease were more specifically investigated in the current translational substudy. TTPBE and TS12 are two endpoints which are objectively associated with the direct effect of BE. In both cases, a series of key predictive markers at 24h were enriched within the KEGG pathway “Pathways in cancer”. This pathway appears to play an important role both in the immediate effect of BE as measured in the blood, and in the prediction of the response to BE.

Our findings could be validated using two independent datasets (meta-analysis from the KMplotter web tool and external CIT validation dataset). The combination of the key predictive markers at 24h regarding TTPBE into a metagene was used to generate a gene signature, predicting with high significance patients into high vs. low risk populations. This signature was successfully validated, and could be used independently from the patient’s EGFR mutational status for proper patient selection.

Because our gene signature is independent from the patient’s mutational status, it can be used as predictive marker both in EGFR mutated and wild-type populations. BE has potential to become a standard therapy in NSCLC patients with EGFR mutations, and our signature may help to select patients which may not respond to the therapy despite the presence of the mutation. Inversely, our signature may be useful for proper selection of BE responders among patients not harboring EGFR activating mutation.

Our findings based on exon array data are in essence exploratory and future prospective confirmatory studies are needed to further validate the clinical relevance of our discovery.

## Conclusion

The 24h effect of BE could be accurately monitored in peripheral blood using the exon array technology. Genes impacted by the immediate effect of BE belonged to key signaling pathways, according to the expected mechanisms of action of both bevacizumab and erlotinib. Although the magnitude of change over 24h had no predictive value with regard to the investigated endpoints, the blood gene expression level measured 24h after initiation of BE could be used to predict TTPBE independently from the patient’s mutational status. Proper selection of responders to the combined targeted therapy BE could be monitored from blood level gene expression.

## Additional file

**Additional file 1.**  R extension package dcca.

## Abbreviations

ABC: ATP binding cassette
ADAM19: ADAM metallopeptidase domain 19
ATAD2: ATPase family, AAA domain containing 2
ATPase: adenosine triphosphatase
B: bevacizumab
BE: bevacizumab/erlotinib
BIRC3: baculoviral IAP repeat containing 3
BMX: BMX non-receptor tyrosine kinase
CASC1: cancer susceptibility candidate 1
CASC5: cancer susceptibility candidate 5
CDC42: cell division cycle 42
CENPE: centromere-associated protein E
CI: confidence interval
CIT: Carte d’Identité des Tumeurs
CT: chemotherapy
DAPK2: death-associated protein kinase 2
DCCA: dually constrained correspondence analysis
DOCK10: dedicator of cytokinesis 10
E: erlotinib
E2F1: E2F transcription factor 1
EGF: epidermal growth factor
EGFR: epidermal growth factor receptor
EKSG: ethics committee of the canton of St. Gallen
EPS8: epidermal growth factor receptor pathway substrate 8
FGFR: fibroblast growth factor receptor
FLT3: Fms-related tyrosine kinase 3
FRY: protein furry homolog
FZD5: frizzled class receptor 5
GBP4: guanylate binding protein 4
GEO: gene expression omnibus
GTPase: guanosine triphosphatase
HR: hazard ratio
IGF1R: insulin-like growth factor 1 receptor
IL1R1: interleukin 1 receptor, type I
IL1R2: interleukin 1 receptor, type II
IQR: inter-quartile range
KEGG: Kyoto encyclopedia of genes and genomes
JUP: junction plakoglobin
KNTC1: kinetochore associated 1
MAPK: mitogen-activated protein kinase
MITF: microphthalmia-associated transcription factor
MME: membrane metalloendopeptidase
MMP9: matrix metallopeptidase 9
mTOR: mechanistic target of rapamycin
NSCLC: non-small cell lung cancer
OS: overall survival
PCA: principal component analysis
PFS: progression-free survival
PLD1: phospholipase D1
RAD51: RAD51 recombinase
RBL1: retinoblastoma-like 1
RNA: ribonucleic acid
SAKK: schweizerische Arbeitsgemeinschaft für klinische Krebsforschung (Swiss Group for Clinical Cancer Research)
TKI: tyrosine kinase inhibitor
TS12: tumor shrinkage at 12 weeks
TTPBE: time to progression under bevacizumab/erlotinib
TTPCT: time to progression under chemotherapy
VEGF: vascular endothelial growth factor
