# Supplementary material for: 24h-gene variation effect of combined bevacizumab/erlotinib in advanced non-squamous non-small cell lung cancer using exon array blood profiling
Source: J Transl Med. 2017 Mar 30;15:66. doi: 10.1186/s12967-017-1174-z (PMC5372268; doi:10.1186/s12967-017-1174-z)
Supplement: Supplementary file 1 — Additional file 1. R extension package dcca. [file 12967_2017_1174_MOESM1_ESM.pdf]

Appendix
24h-gene variation effect of combined bevacizumab/erlotinib in advanced non-squamous non-small cell lung cancer using exon array blood profiling

Florent Baty, Markus Jörger, Martin Früh, Dirk Klingbiel, Francesco Zappa, Martin Brutsche

Methods

Ordinary correspondence analysis

The core procedure in DCCA is correspondence analysis (CA), a powerful ordination method classically used for the analysis of contingency tables [1], and more generally applicable for the analysis of tables of positive or null values (e.g. genomics data) [2]. Ordinary correspondence analyses are used to investigate the dependence between rows and columns in a data set. Theoretical basis underlying CA can be summarized by defining the following:

- $\mathbf{X}$ the $n \times m$ matrix of exon-level expression data (n samples, m exons)
- $\mathbf{P} = \mathbf{X}/N$ the data matrix divided by its grand total
- $\mathbf{r}$ the $n-$dim vector of row sums of $\mathbf{P}$ (row weights)
- $\mathbf{c}$ the $m-$dim vector of column sums of $\mathbf{P}$ (column weights)
- $\mathbf{D}_r$ the $n \times n$ diagonal matrix of row sums
- $\mathbf{D}_c$ the $m \times m$ diagonal matrix of column sums

In correspondence analysis, the main matrix of interest is converted into a χ^2 distance matrix after the following pre-processing data transformation:

$$\mathbf{Z} = \mathbf{D}_r^{-1/2}(\mathbf{P} - \mathbf{r}\mathbf{c}^T)\mathbf{D}_c^{-1/2} \tag{1}$$

Correspondence analysis performs the singular value decomposition of $\mathbf{Z}$:

$$\mathbf{Z} = \mathbf{U}\mathbf{\Lambda}\mathbf{V}^T \tag{2}$$

with $\mathbf{\Lambda}$ the $k \times k$ ($k = rank(\mathbf{Z})$) diagonal matrix of singular values associated with $\mathbf{Z}$ with $\lambda_1 \geq \ldots \geq \lambda_k > 0$, $\mathbf{U}$ an $n \times k$ matrix whose columns are the left singular vectors of $\mathbf{Z}$ and $\mathbf{V}$ an $m \times k$ matrix whose columns are the right singular vectors of $\mathbf{Z}$. The rows of $\mathbf{U}$ and $\mathbf{V}$ are orthonormal with respect to $\mathbf{D}_r$ and $\mathbf{D}_c$, respectively:

$$\mathbf{U}^T\mathbf{D}_r\mathbf{U} = \mathbf{V}^T\mathbf{D}_c\mathbf{V} = \mathbf{I} \tag{3}$$

The principal components and row coordinates are given by $\mathbf{D}_r^{-1/2}\mathbf{U}$ and $\mathbf{D}_r^{-1/2}\mathbf{U}\mathbf{\Lambda}$, respectively. The principal axes and column coordinates are given by $\mathbf{D}_c^{-1/2}\mathbf{V}$ and $\mathbf{D}_c^{-1/2}\mathbf{V}\mathbf{\Lambda}$, respectively.

Three-way correspondence analysis

Experimental designs including a repeated measurement of all variables for all subjects at a second time-point imply 3 dimensional data sets (patient $\times$ exons $\times$ time). In the current setting, a pair of tables fully matched on rows and columns was considered. Several approaches have been proposed for the analysis of these specific data in the framework of correspondence analysis. These include methods directly dealing with three-dimensional structures [3], and a series of methods unrolling the third dimension of the data into data structures that can be handled by conventional 2-dimensional CA, such as Foucart's CA [4] or STATIS-CoA [5]. However, these methods generally focus on the similarity of the matched tables (by means of a consensus matrix) rather than on their differences. Our data specifically contain 2 exon-array data sets measuring the same set of exon-level expressions for the same patients, before and 24h after initiation of the treatment. In this situation where the 2 data sets are fully matched, the main question of interest is the identification of variations related to the immediate treatment effect. We are interested in analyzing the expression changes measured at 24h, taking the expression at baseline as reference. The analysis should properly take into account the within-patient experimental design. A few solutions to this problem have been proposed [6, 7, 8]. The simplest procedure proposed by Torre & Chessel [7], and used in the current work, consists in staking observation-wise the 2 matrices into one table, and carrying out a within-class analysis by defining a categorical variable describing each pair of samples. In this analysis, the within-patient design is accounted for by partialling out the patient effect, which enables to directly investigate the differences before vs. after treatment.

Dually constrained correspondence analysis

Incorporating external constraints in CA is desirable for the direct interpretation of CA in the light of external information structuring the rows and/or columns of a data set. DCCA is an extension of ordinary CA where 2 sets of linear constraints are applied on both rows and columns. Two complementary approaches can be used to impose constraints in the analysis: the reparametrization method and the null space method [9].

Positive constraints can be applied row-wise and column-wise using the respective projection operators:

$$\mathbf{O}_r = \mathbf{M}(\mathbf{M}^T\mathbf{D}_r\mathbf{M})^{-1}\mathbf{M}^T\mathbf{D}_r \tag{4}$$

$$\mathbf{O}_c = \mathbf{N}(\mathbf{N}^T\mathbf{D}_c\mathbf{N})^{-1}\mathbf{N}^T\mathbf{D}_c \tag{5}$$

Negative constraints can be applied row-wise and column-wise using the respective orthogonal projection operators:

$$\mathbf{Q}_r = \mathbf{I} - \mathbf{G}(\mathbf{G}^T\mathbf{D}_r^{-1}\mathbf{G})^{-1}\mathbf{G}^T\mathbf{D}_r^{-1} \tag{6}$$

$$\mathbf{Q}_c = \mathbf{I} - \mathbf{H}(\mathbf{H}^T\mathbf{D}_c^{-1}\mathbf{H})^{-1}\mathbf{H}^T\mathbf{D}_c^{-1} \tag{7}$$

Notice that positive and negative constraints can be combined by examining the effect of one set of variable $\mathbf{X}_2$ while statistically controlling for the effects of a second set of variables $\mathbf{X}_1$. This is done by partialling out the effect of $\mathbf{X}_1$ from $\mathbf{X}_2$ using the reparametrization method. The resulting row-wise and column-wise partial constraints are the following:

$$\mathbf{O}_r^* = \mathbf{M}^*(\mathbf{M}^{*T}\mathbf{D}_r\mathbf{M}^*)^{-1}\mathbf{M}^{*T}\mathbf{D}_r \tag{8}$$

with $\mathbf{M}^* = (\mathbf{I} - \mathbf{X}_1(\mathbf{X}_1^T\mathbf{D}_r\mathbf{X}_1)^{-1}\mathbf{X}_1^T\mathbf{D}_r)\mathbf{X}_2$

$$\mathbf{O}_c^* = \mathbf{N}^*(\mathbf{N}^{*T}\mathbf{D}_c\mathbf{N}^*)^{-1}\mathbf{N}^{*T}\mathbf{D}_c \tag{9}$$

with $\mathbf{N}^* = (\mathbf{I} - \mathbf{X}_1(\mathbf{X}_1^T\mathbf{D}_c\mathbf{X}_1)^{-1}\mathbf{X}_1^T\mathbf{D}_c)\mathbf{X}_2$

Notice that when applied to the observations only, this type of constraints corresponds to the partial canonical correspondence analysis described by ter Braak [10].

Depending on the study objective three types of constraints can be applied row-wise and columns-wise. The last step consists in performing one of the 9 possible generalized singular value decompositions:

$$
\mathbf{Z}^* = \begin{cases}
\mathbf{D}_r^{-1/2}\mathbf{O}_r(\mathbf{P}-\mathbf{r}\mathbf{c}^T)\mathbf{O}_c^T\mathbf{D}_c^{-1/2} = \mathbf{U}^*\boldsymbol{\Lambda}^*\mathbf{V}^{*T} \\
\mathbf{D}_r^{-1/2}\mathbf{O}_r(\mathbf{P}-\mathbf{r}\mathbf{c}^T)\mathbf{Q}_c^T\mathbf{D}_c^{-1/2} = \mathbf{U}^*\boldsymbol{\Lambda}^*\mathbf{V}^{*T} \\
\mathbf{D}_r^{-1/2}\mathbf{O}_r(\mathbf{P}-\mathbf{r}\mathbf{c}^T)\mathbf{O}_c^{*T}\mathbf{D}_c^{-1/2} = \mathbf{U}^*\boldsymbol{\Lambda}^*\mathbf{V}^{*T} \\
\mathbf{D}_r^{-1/2}\mathbf{Q}_r(\mathbf{P}-\mathbf{r}\mathbf{c}^T)\mathbf{O}_c^T\mathbf{D}_c^{-1/2} = \mathbf{U}^*\boldsymbol{\Lambda}^*\mathbf{V}^{*T} \\
\mathbf{D}_r^{-1/2}\mathbf{Q}_r(\mathbf{P}-\mathbf{r}\mathbf{c}^T)\mathbf{Q}_c^T\mathbf{D}_c^{-1/2} = \mathbf{U}^*\boldsymbol{\Lambda}^*\mathbf{V}^{*T} \\
\mathbf{D}_r^{-1/2}\mathbf{Q}_r(\mathbf{P}-\mathbf{r}\mathbf{c}^T)\mathbf{O}_c^{*T}\mathbf{D}_c^{-1/2} = \mathbf{U}^*\boldsymbol{\Lambda}^*\mathbf{V}^{*T} \\
\mathbf{D}_r^{-1/2}\mathbf{O}_r^*(\mathbf{P}-\mathbf{r}\mathbf{c}^T)\mathbf{O}_c^T\mathbf{D}_c^{-1/2} = \mathbf{U}^*\boldsymbol{\Lambda}^*\mathbf{V}^{*T} \\
\mathbf{D}_r^{-1/2}\mathbf{O}_r^*(\mathbf{P}-\mathbf{r}\mathbf{c}^T)\mathbf{Q}_c^T\mathbf{D}_c^{-1/2} = \mathbf{U}^*\boldsymbol{\Lambda}^*\mathbf{V}^{*T} \\
\mathbf{D}_r^{-1/2}\mathbf{O}_r^*(\mathbf{P}-\mathbf{r}\mathbf{c}^T)\mathbf{O}_c^{*T}\mathbf{D}_c^{-1/2} = \mathbf{U}^*\boldsymbol{\Lambda}^*\mathbf{V}^{*T}
\end{cases} \quad (10)
$$

with $\mathbf{U}^{*T}\mathbf{D}_r\mathbf{U}^* = \mathbf{V}^{*T}\mathbf{D}_c\mathbf{V}^* = \mathbf{I}$

The principal components and row coordinates are given by $\mathbf{D}_r^{-1/2}\mathbf{U}^*$ and $\mathbf{D}_r^{-1/2}\mathbf{U}^*\boldsymbol{\Lambda}^*$, respectively. The principal axes and column coordinates are given by $\mathbf{D}_c^{-1/2}\mathbf{V}^*$ and $\mathbf{D}_c^{-1/2}\mathbf{V}^*\boldsymbol{\Lambda}^*$, respectively.

The contribution of the jth variable to the lth dimension in DCCA is expressed as follows:

$$
\text{ctr}_{j,l} = c_j \times q_{j,l}^2 \quad (11)
$$

with c_j the weight of the jth variable, and $q_{j,l}^2$ the coordinate of the jth variable (loading) on the lth dimension (principal axes).

Design of experiment

The structure of the current data set and the scheme of DCCA are summarized in Figure 1.

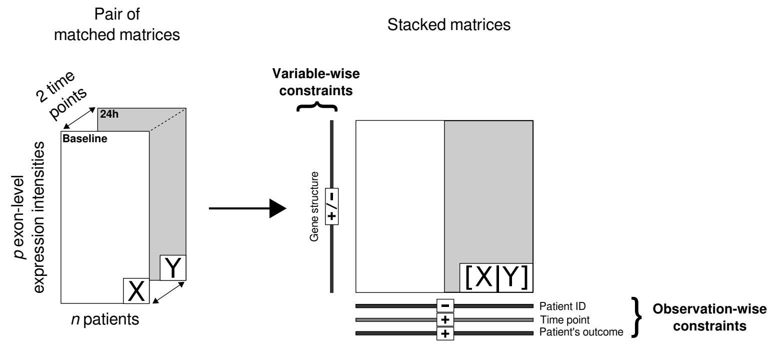

Figure 1: Design of experiment and scheme of Dually Constrained Correspondence Analysis. Two matched tables $\mathbf{X}$ and $\mathbf{Y}$ are analyzed by DCCA. The 2 tables are rearranged into one stacked table. Additional external information on both rows and columns are used as positive and/or negative constraints.

The CA model is dually constrained. Variable-wise, a categorical variable indicating which exon belongs to which gene is used either as a positive constraint (gene-level analysis) or as a negative constraint (exon-level/alternative splicing analysis). Observation-wise, two categorical variables are defined, corresponding to the patient identifier and the time when the measurement was made. Following the previous annotations, the following two transformations are considered:

$$\mathbf{Z}^* = \mathbf{D}_r^{-1/2}\mathbf{O}_r^*(\mathbf{P} - \mathbf{r}\mathbf{c}^T)\mathbf{O}_c^T\mathbf{D}_c^{-1/2} \tag{12}$$

and

$$\mathbf{Z}^* = \mathbf{D}_r^{-1/2}\mathbf{O}_r^*(\mathbf{P} - \mathbf{r}\mathbf{c}^T)\mathbf{Q}_c^T\mathbf{D}_c^{-1/2} \tag{13}$$

for the gene-level and exon-level analysis, respectively.

Results

Selection of responders based the metagene score

An illustration of selection of responders based the metagene score is given in Figure 2. Figure 2 depicts the time to progression under BE as a function of the metagene score. Each dot represent a patient either showing a progression (plain dot) or censored (empty dot). The red color indicate the presence of characterized EGFR mutations. Patients with a high metagene score showed a better response to BE. All EGFR mutated patients (red dots) had a TTPBE above the median time to progression. However, as shown in the upper right corner of the plot, several patients without characterized EGFR mutation showed a satisfactory response to BE.

References

[1] Benzécri, J.P.: L'analyse des Données: L'analyse des Correspondances. Dunod, Paris (1973)

[2] Fellenberg, K., Hauser, N.C., Brors, B., Neutzner, A., Hoheisel, J.D., Vingron, M.: Correspondence analysis applied to microarray data. Proc. Natl. Acad. Sci. U.S.A. **98**(19), 10781–10786 (2001)

[3] Carlier, A., Kroonenberg, P.M.: Decompositions and biplots in three-way correspondence analysis. Psychometrika **61**, 355–373 (1996)

[4] Pavoine, S., Blondel, J., Baguette, M., Chessel, D.: A new technique for ordering asymmetrical three-dimensional data sets in ecology. Ecology **88**(2), 512–523 (2007)

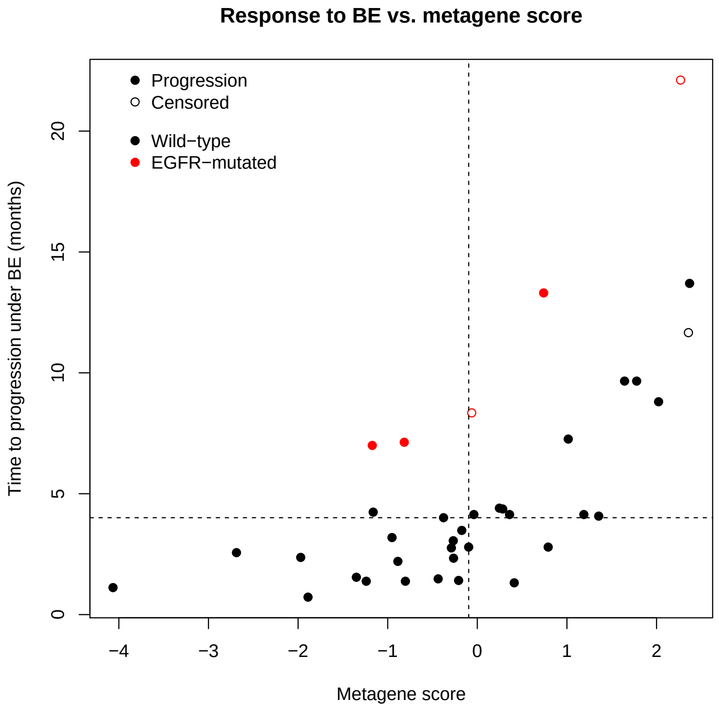

Figure 2: Prediction of the response to BE using the metagene score. The figure is a representation of the time to progression under BE as a function of the metagene score. Patients with progression (plain dots) or censored (empty dots), wild type (black dots) or harboring EGFR-mutations (red dots) are displayed. The horizontal and vertical dashed lines represent the median time to progression and the median metagene score, respectively.

[5] Gaertner, J.C., Bertrand, J., Souplet, A.: Statis-coa: A methodological solution to assess the spatio-temporal organization of species assemblages. application to the demersal assemblages of the french mediterranean sea. Scientia Marina **66**(S2) (2002)

[6] Greenacre, M.J.: Singular value decomposition of matched matrices. Journal of Applied Statistics **30**(10), 1101–1113 (2003)

[7] Torre, F., Chessel, D.: Co-structure de deux tableaux totalement apparis. Rev. Statistique Applique **43**, 109–121 (1995)

[8] Lafosse, R.: Ressemblance et diffé'rence entre deux tableaux totalement appariés. Rev. Statistique Appliquée **43**, 109–121 (1995)

[9] Böckenholt, U., Takane, Y.: Linear constraints in correspondence analysis. In: Greenacre, M., Blasius, J. (eds.) Correspondence Analysis in Social Sciences, pp. 112–127. Academic Press, New York (1993)

[10] ter Braak, C.J.F.: Partial canonical correspondence analysis. In: Bock, H.H. (ed.) Classification and Related Methods of Data Analysis, pp. 551–558. Elsevier Science Publishers, Amsterdam (1988)
